# Shiitake Mushroom Dermatitis in a Returning Traveler

**DOI:** 10.4269/ajtmh.19-0882

**Published:** 2020-02-05

**Authors:** Henrietta Mills, Stephen L. Walker

**Affiliations:** Hospital for Tropical Diseases; University College London Hospitals NHS Foundation Trust; London, United Kingdom; E-mail: henriettamills@gmail.com; Hospital for Tropical Diseases and Department of Dermatology; University College London Hospitals NHS Foundation Trust; London, United Kingdom; Faculty of Infectious and Tropical Diseases; London School of Hygiene and Tropical Medicine; London, United Kingdom; E-mail: steve.walker@Lshtm.ac.uk

Dear Editor,

We agree with the editorial by Philip J. Rosenthal “Be Careful What You Eat!”^1^ and wish to highlight other foodstuffs which can have a deleterious impact on the health of returning travelers.

A 30-year-old woman attended our emergency clinic with a 2-day history of a widespread burning and pruritic rash following a trip to Hong Kong, where she had sustained multiple arthropod bites on the lower limbs. She had a history of well-controlled atopic dermatitis and, although she had witnessed the current public disorder, had had no direct contact with tear gas.

**Figure 1.**
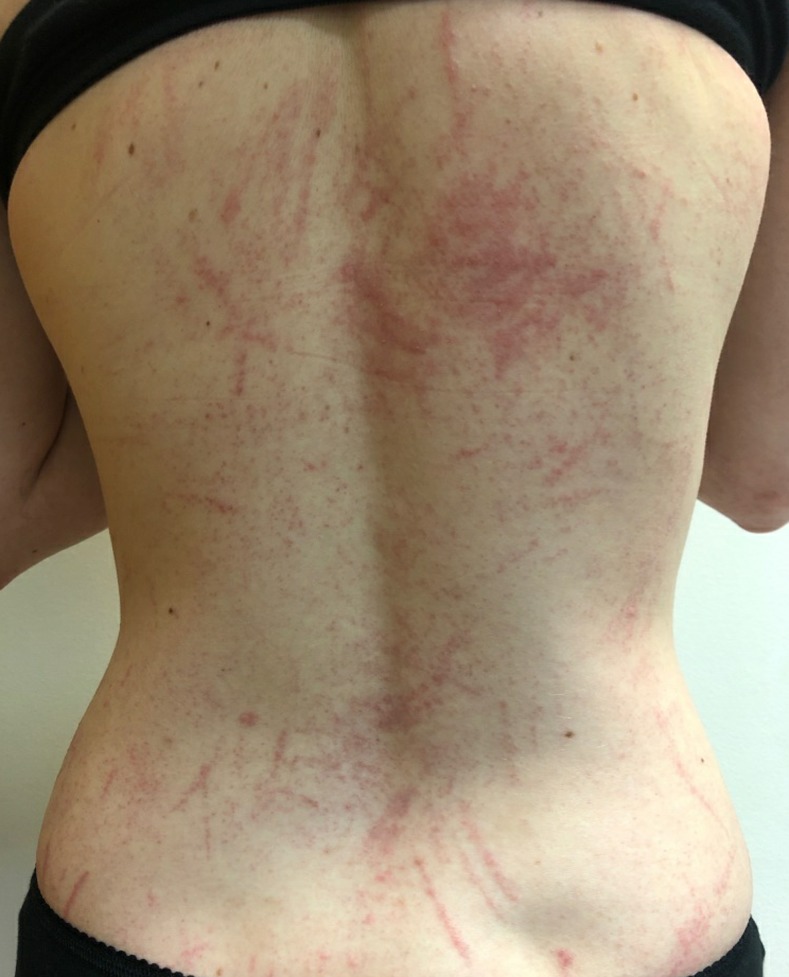
Flagellate dermatitis due to shiitake mushroom dermatitis. This figure appears in color at www.ajtmh.org.

On examination, she was afebrile. There was a linear, erythematous, and slightly raised eruption on the torso and limbs (Figure 1), suggestive of scratch marks, with erythematous papules on the hands, legs, and feet. The face was spared. There was a patch of atopic dermatitis in each antecubital fossa, but no dermographism. There was no peripheral eosinophilia, C-reactive protein was normal, and HIV, syphilis, and antistreptolysin O serologies were negative.

The appearance of the rash caused us to take a detailed dietary history. She was a vegan and had eaten four skewers of shiitake mushrooms purchased from a street vendor approximately 48 hours before the onset of her symptoms. A diagnosis of shiitake mushroom dermatitis was made, and she was treated with oral prednisolone, 30 mg daily for 5 days, with complete resolution of her symptoms.

A flagellate rash following the recent ingestion of shiitake mushrooms (*Lentinus edodes*) is diagnostic.^2^ The dermatitis is a toxic reaction to a thermolabile polysaccharide, lentinan, which is present in raw and undercooked shiitake mushrooms.^2^ The rash can appear from two hours to five days following ingestion and may take up to 28 days to resolve.^2^ Severe cutaneous reactions have been reported.^3^

This case highlights the importance of a thorough dietary history in returning travelers with unexplained symptoms and that being “careful what you eat” is not restricted to raw or undercooked animal products.
